# A TEAM APPROACH TO THE IMPLEMENTATION OF THE ANNUAL WELLNESS VISIT

**DOI:** 10.1093/geroni/igz038.1447

**Published:** 2019-11-08

**Authors:** Lucy Guerra

**Affiliations:** University of Florida, Tampa, Florida, United States

## Abstract

The success of any practice change initiative is dependent upon a highly effective team. This presentation will focus on the “secret sauce” of our implementation of the AWV in collaboration with the Dartmouth GWEP. Participating in both asynchronous and live virtual training enabled our team to come together to successfully implement the AWV using a team based model. Using the principles of highly effective teaming and a rapid cycle QI approach we have been able to improve patient outcomes in primary care.

